# Method of Remedying Accidents Caused by Chloroform

**Published:** 1852-12

**Authors:** 


From the Boston Medical Journal.

*Method of Remedying Accidents caused by Chloroform.*

A letter from M. Ricord was published in the Journal de Chimie in January, 1850, in which he describes a simple method practised by him in cases of serious effects from the use of chloroform. He gives the particulars of two cases in which the method was successful. These we copy from the London Lancet.

"Case 1.—The patient who furnishes the subject of my first case, was a woman of about twenty-six, from which I was about to remove some growths of no great size. She was previously chloroformed, to which she only submitted after repeated entreaties, for she appeared to be excessively timid.

"The anæsthetic effect of the chloroform was very rapid, for after a few respirations she appeared asleep; the sponge was removed, and I commenced excising the growths, but had scarcely given two or three cuts, when one of my assistant surgeons told me that the pulse appeared to be failing. I now saw, in fact, that the beating of the heart was suspended, that all respiratory movements had ceased, and that the lips were livid, and hung down. The limbs were completely relaxed, and the paleness of the face showed that the patient was in that state of syncope which is the herald of death. All the remedies indicated in such a case were forthwith employed, as cold currents of air, sprinkling cold water on the face, tickling the nostrils, &c. Artificial respiration, by pressure on the walls of the chest, was tried.

"The syncope continued, and death seemed close at hand. I began to be uneasy, and determined to try direct insufflation. I applied my mouth to that of the patient. After some inspirations the dying woman gave a sigh, her chest heaved, the face resumed its normal color, the heart and pulse commenced beating in an appreciable manner, and the eyes opened; respiration had again brought into play all the functions of life, and the return of sensation was evinced by a smile. The patient was saved, and we escaped with the fright.

"Case 2.—The second time that I experienced the dangers of chloroform was with a patient under my care in the Southern Hospital (Hopital du Midi.) He was a young man whose case required circumcision. As this operation is generally painful enough, he asked me to send him to sleep with the chloroform. A sponge impregnated with it was given him to respire from: the action was very rapid, without any appearance of preceding excitement, and the patient was soon plunged in total insensibility. I performed the operation, but when it was concluded, the patient did not recover his consciousness, and remained in a state of alarming stillness. The pulse gradually sunk; the heart ceased to beat: all the sphincters were relaxed, and his cadaverous face seemed to testify that death was near.

"All the means I have indicated in the preceding case were tried but without avail, and it became necessary to have recourse to insufflation, which had already so well succeeded in one case. Success crowned my efforts, and the patient recovered."

